# The Regulatory Role of Activating Transcription Factor 2 in Inflammation

**DOI:** 10.1155/2014/950472

**Published:** 2014-06-23

**Authors:** Tao Yu, Yong Jun Li, Ai Hong Bian, Hui Bin Zuo, Ti Wen Zhu, Sheng Xiang Ji, Fanming Kong, De Qing Yin, Chuan Bao Wang, Zi Fu Wang, Hong Qun Wang, Yanyan Yang, Byong Chul Yoo, Jae Youl Cho

**Affiliations:** ^1^Linyi Center for Disease Control and Prevention, Linyi 276000, China; ^2^Department of Genetic Engineering, Sungkyunkwan University, Suwon 440-746, Republic of Korea; ^3^Laiwu Center for Disease Control and Prevention, Laiwu 271100, China; ^4^Research Institute and Hospital, National Cancer Center, Goyang 410-769, Republic of Korea

## Abstract

Activating transcription factor 2 (ATF2) is a member of the leucine zipper family of DNA-binding proteins and is widely distributed in tissues including the liver, lung, spleen, and kidney. Like c-Jun and c-Fos, ATF2 responds to stress-related stimuli and may thereby influence cell proliferation, inflammation, apoptosis, oncogenesis, neurological development and function, and skeletal remodeling. Recent studies clarify the regulatory role of ATF2 in inflammation and describe potential inhibitors of this protein. In this paper, we summarize the properties and functions of ATF2 and explore potential applications of ATF2 inhibitors as tools for research and for the development of immunosuppressive and anti-inflammatory drugs.

## 1. Introduction

Inflammation functions as a subcomponent of the immune response, which is a system of cells and tissues that evolved to maintain the boundaries between specialized cell populations and tissues of multicellular organisms. Immunity and inflammation block invasion by microbial pathogens and contribute to an organism's response to stress. Pathological effects may result from a prolonged or abnormal inflammatory response, as in asthma and autoimmunity [1, 2]. Locally, inflammation evokes redness, swelling, heat, and pain, as sensitized cells attack the foreign cells with soluble mediators (cytokines) and/or engulf the stress-inducing agent. Systemic signs of inflammation include fever and increasing numbers of bone marrow- and thymus-derived white blood cells, such as macrophages and lymphocytes [3, 4]. The local vascular component of inflammation forms a critical link with the systemic immune defense or stress response. In the process of inflammation, molecular mediators may directly and/or indirectly injure normal cells and tissues [5, 6]. Although tissue congestion and exudates may dilute and degrade cytotoxic factors, the underlying stromal cells gradually regenerate to repair and heal damaged tissues. The inflammatory response may therefore be described as a dynamic process of demolition and repair.

Inflammation is categorized into acute and chronic inflammation based on duration. Acute inflammation, with redness, swelling, and pain, is a short-term process that reflects the vascular component. In chronic inflammation, such as autoimmune disease and tuberculosis, soluble mediators persist at lower levels than in acute inflammation but for longer periods; cells chiefly involved include lymphocytes, plasma cells, and macrophages. The macrophages are differentiated from mononuclear precursors through the influence of specific factors, such as granulocyte-macrophage colony-stimulating factor (GM-CSF), and nonspecific agents, such as phorbol-12-myristate-13-acetate (PMA). Macrophages participate in both innate and adaptive immune processes. Phenotypically, macrophages and monocytes are phagocytic white blood cells [7], which are able to recognize, engulf, and digest cellular debris and pathogens [8]. Macrophages interact with the environment through a diverse array of receptors expressed at the plasma membrane [9–11]. When macrophages bind to and recognize a microorganism, abnormal cell, or immunogenic chemical, they undergo a complex phenotypic transformation that leads to recruitment and activation of other cell types involved in innate or acquired immunity [12]. Depending on the stimulus and the cells recruited, different compartments of the immune system may be drawn into the defense [13].

Activating transcription factor 2 (ATF2) is a transcription factor of the leucine zipper family of DNA-binding proteins, discovered in 1991 by Ozawa et al. [46] and located on human chromosome 2q32. The ATF2 protein consists of 505 amino acids, with phosphorylation sites near the C-terminus at serine residues 472 and 480 in the mouse protein and serines 490 and 498 in the human protein. In response to double-stranded DNA breaks, the ataxia telangiectasia-mutant (ATM) protein kinase activates ATF2 [47]. The ATF family of proteins includes six subtypes based on sequence similarity [48]. ATF proteins play critical roles in cell proliferation, apoptosis, inflammation, and cancer.

In this study, we describe the general properties of ATF2, with particular emphasis on its role in inflammation. In addition, we review recently identified ATF2 inhibitors, including naturally occurring compounds, plant extracts, and gene expression inhibitors, which have potential applications in the treatment of inflammatory diseases.

## 2. General Features of ATF Family Proteins

### 2.1. ATF Family Proteins

**Figure 1:**
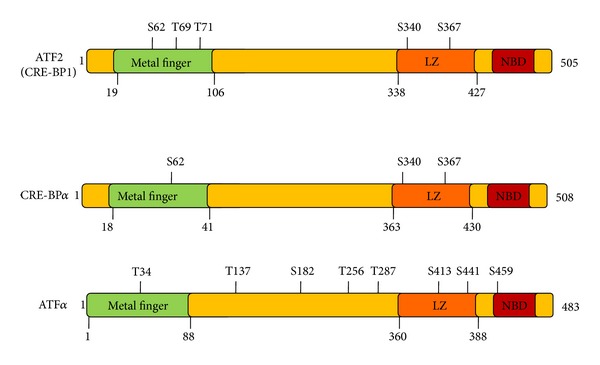
Characteristics of ATF proteins. ATF: activating transcription factor; LZ: leucine zipper; NBD: nucleotide-binding domain.

The ATF/CREB family consists of six subtypes based on sequence similarity, including CREB, CRE-BP1 (ATF2), ATF3, ATF4, ATF6, and B-ATF [49]. These all share the common bZIP element, through which they dimerize and bind to the palindromic cAMP response element (CRE) octanucleotide TGACGTCA in DNA [49]. The N-terminal domains of ATF proteins show divergence, but the C-terminal leucine zipper for dimerization and DNA binding is well conserved. The ATF2 group, which was originally designated CRE-BP1, contains ATF2, CRE-BP*α*, and ATF7 (also known as ATF*α*) (Figure 1). Close sequence similarity sets these proteins apart as a subgroup, which is distinguished by the metal finger structure and the leucine zipper structure in the NH_2_- and COOH-terminal regions; these motifs are essential for transactivation [34, 50–52]. However, despite strong sequence similarity, the ATF2 proteins differ in function, phosphorylation sites, and expression patterns [50]. CRE-BP1 is detected in most cells and tissues but is especially abundant in brain and regenerating liver [50, 53]. CRE-BP*α* is detected in a limited number of cell lines and tissues, including HeLa cells and the placenta [50]. ATF*α* is ubiquitously expressed in fetal and adult mice, with high expression in squamous epithelia and brain tissue [54]. The ATF2 protein resides as a homodimer in the cytoplasm but is retained as a heterodimer with Jun in the nucleus [55]. Normally, ATF2 localizes in the cytoplasm at the mitochondrial outer membrane [56]. The characteristics of ATF family proteins are summarized in Table 1.

### 2.2. ATF2 Activation Signaling Pathways

**Figure 2:**
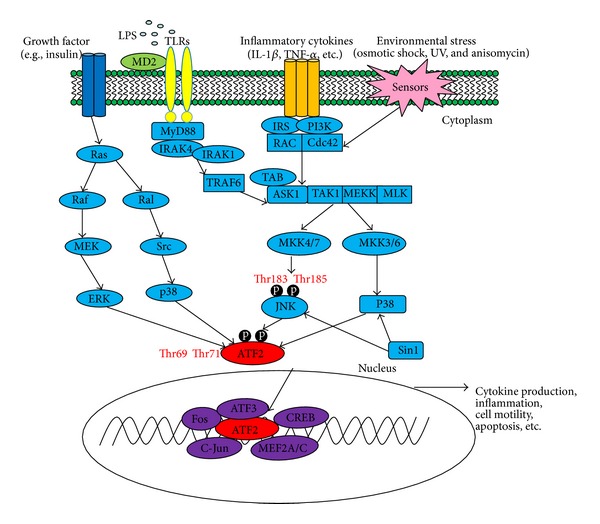
ATF-2-regulated signaling pathways in inflammatory response.

Hormones are essential for the maintenance of regenerative tissues. They include the endocrine hormones, which circulate systemically, and a diverse population of growth factors, which are locally active proteins produced by specific cell types. The growth factor proteins may be further characterized as paracrine factors, which act between cells, and autocrine factors, which act within the cells that produce them. The endocrine hormones include both proteins and steroids, which are distinguished by the mode of signal transduction engaged. Steroid hormones such as estrogen pass through the cell membrane, bind to specific cytoplasmic receptors, and travel to the nucleus to interact directly with target genes. Protein hormones such as insulin and epidermal growth factor (EGF) bind to extracellular membrane receptors, triggering an intracellular cascade of enzymatic signaling reactions leading to changes in nuclear gene expression. Proinflammatory signaling may be activated specifically by hormonal and cellular mechanisms. Nonspecific substances such as allergens, antigens, and irritating substances can also activate proinflammatory signaling. After such stimulation, ATF2 may be activated by two alternative Ras-coupled pathways [57]. Through the Raf-MEK-ERK pathway, threonine 71 of ATF2 is phosphorylated. Through the Ral-RalGDS-Src-p38 pathway, Thr69 is phosphorylated (Figure 2). In growth factor-activated cells, p38 and JNK mediation of phosphorylation at Thr71 or Thr69+71 cannot account for the level of ATF2 activation observed, nor can ERK-mediated phosphorylation of Thr69+71 alone activate ATF2 efficiently [57].

Many environmental stressors, including ultraviolet light, heat shock, osmotic stress, and oxidative stress, may activate ATF2, usually through stress sensors linked to downstream target proteins in the Rac/cdc42 pathway. Through this pathway, the MEKKs/MLKs-SEK/MKKs-JNK/p38 pathway may be activated, leading to ATF2 phosphorylation at Thr69 and Thr71 sites without involvement of ERK [49, 58–64] (Figure 2).

## 3. Functional Involvement of ATF2 in Inflammation

ATF proteins are widely investigated as procarcinogenic factors in tumors of the prostate, breast, liver, and lung, as well as in leukemia [49]. Specific roles for these proteins in inflammatory diseases are also emerging. For example, ATF1 is involved in wound healing and gingival inflammation [65]. ATF3 attenuates transcription of the proinflammatory gene MCP-1 in renal ischemia-reperfusion injury [14] and may significantly influence inflammatory processes in the central nervous system (CNS) that are related to the frequency of epileptic seizures [66]. Recent evidence suggests a role for ATF2 in inflammation. Reports have demonstrated that ATF2 is highly expressed in infiltrating macrophages and may suppress ATF3 transcription in M1 macrophages of white adipose tissues in obesity [67]. ATF3 may be a nuclear mediator of inflammatory pain that is induced through the p38-mitogen-activated protein kinase (MAPK) signaling pathway [68]. ATF2 is observed as a serological marker for inflammation and lung involvement in systemic sclerosis (SSc) [69]. In LPS-induced hepatitis and HCl/EtOH-induced gastritis, we recently observed significant activation of ATF2 (unpublished data), suggesting that ATF2 participates in these inflammatory processes [35]. Furthermore, the death rate of ATF2-mutant mice was much higher than that of the control group when treated with LPS and D-galactosamine, implying that ATF2 may play a critical role in LPS-induced toxicity [70].

### 3.1. ATF2-Activated Proinflammatory Genes

Activated ATF2 complexes stimulate the transcription of various genes implicated in inflammation such as cell adhesion molecules (CAMs), proinflammatory cytokines, and chemokines. CAMs expressed on cell surfaces engage in binding with other cells or with the extracellular matrix (ECM). CAM proteins include the integrins, cadherins, and selectins (E-selectin, P-selectin, and L-selectin). Selectins participate in the initial recruitment of leukocytes to the site of injury during inflammation. VCAM-1 may influence the development of atherosclerosis and rheumatoid arthritis. In ATF2-deficient mice, the induction of E-selectin, P-selectin, and VCAM-1 in lung and kidney following lipopolysaccharide injection was significantly reduced when compared to control mice; in addition, responses to proinflammatory and infectious challenges were delayed or suppressed [70].

Cells that orchestrate an immune response produce an array of soluble protein factors, or cytokines, to immobilize, kill, sequester, or eliminate invasive cells and microorganisms. Systemic effects of this response include fever, tissue destruction, septic shock, and death [71]. The proinflammatory cytokine TNF-*α*, produced mainly by macrophages, lymphoid cells, mast cells, and adipose tissue, causes a variety of clinical inflammatory disorders such as rheumatoid arthritis, psoriasis, refractory asthma, and inflammatory bowel disease. In ATF2 knockout mice, TNF-*α* expression was significantly inhibited. In addition, interleukin- (IL-) 1*β* and IL-6 were also dramatically suppressed in ATF2-deficient mice [70].

The soluble factor keratinocyte chemoattractants are the most highly inducible chemokines produced by IL-1 and TNF-*α*. They are involved in chemotaxis, cell-mediated activation of neutrophils, and the neutrophil inflammatory responses. Interestingly, in ATF2-deficient mice, the expression of keratinocyte chemoattractants was clearly suppressed [70]. Furthermore, a regulatory role for ATF2 in rennin expression has been also reported [72].

### 3.2. Signaling Pathways for ATF2 Activation in Inflammation

Pattern recognition receptor proteins (PRRs) enable mammalian cells and organisms to recognize invading microorganisms and abnormal or injured cells. The toll-like receptors (TLRs) play an especially important role in the innate immune response by recognizing surface patterns on microbial invaders [73]. Eleven TLR-family proteins have been identified to date [4]. Activation of TLR signaling may be initiated at intracytoplasmic TIR domains, which are conserved among all TLRs. TIR domain-containing adaptor proteins include MyD88, TIRAP, and TRIF. The induction of inflammatory cytokines requires MyD88. In response to specific ligands, MyD88 recruits IL-1 receptor-associated kinase-4 (IRAK4) to the TLRs by interacting with the death domains of both molecules. IRAK-1 is activated by phosphorylation and associates with TRAF6, activating the IKK complex and MAP kinases (JNK, p38, and ERK) [74–77]. ATF2 is maintained in a transcriptionally inactive form through intramolecular interactions between its own activation domain and its bZIP domain [49, 78]. In response to activated upstream signaling factors p38 and JNK, ATF2 is phosphorylated at amino acids Thr69 and Thr71. Phosphorylated ATF2 may then form homodimers or heterodimers with other members of the ATF/CREB family and the Fos/Jun family [49, 79] (Figure 2).

### 3.3. The Role of ATF2 in Inflammation-Derived Disease

*In vitro* studies in human and mouse cell lines as well as knockout mice reveal the activation of ATF2 in several inflammatory diseases including obesity, hepatitis, inflammatory pain, and allergic asthma [80, 81].

#### 3.3.1. Obesity

The white adipose tissue (WAT) that accumulates in obesity displays multiple markers of inflammation, with progressive infiltration by macrophages and generation of reactive oxygen species (ROS). Some evidence links these markers to insulin resistance and adipokine dysregulation [67]. In genetically obese (ob/ob) mice, both total and phosphorylated ATF2 are highly expressed in macrophages infiltrating the WAT. In RAW264.7 macrophages, ATF2 activation may be induced by treatment with either H_2_O_2_ or LPS [67]. In ATF2-mutant mice, WAT is significantly less abundant than in the wild-type mouse [34]. Treatment of mice with inhibitors of p38-ATF2 signaling suppresses adipocyte differentiation and WAT accumulation. In addition, it may counteract high-fat diet (HFD) induced obesity, insulin resistance, macrophage infiltration into WAT, and the associated increase in TNF-*α* expression [34]. Evidence for p38-linked ATF2 signaling as a regulatory component in obesity-related inflammation may lend insight into the pathological effects of overnutrition.

#### 3.3.2. Hepatitis

The most common etiology of liver inflammation in industrialized countries is infection with type A, B, or C viral hepatitis. Globally, about 250 million people are estimated to be infected with hepatitis C, and 300 million may be infected with hepatitis B [82]. Overdose of alcohol and certain drugs may cause hepatitis and intensify the effects of viral infection on liver function. There are conflicting findings on the involvement of ATF2 in hepatic inflammation. On the one hand, ATF2 is reported to suppress activity at the hepatitis B virus X promoter through competition for the activating protein 1 binding site and through formation of a ATF2-Jun heterodimer [83]. However, signaling through the PKA-CREB/ATF2 pathway is reported to activate and maintain transcriptional activity at the hepatitis B virus pre-S2/S promoter [24]. We found that, in mice with hepatitis induced by treatment for 7 days with D-gal/LPS, levels of activated ATF2 were significantly higher than in the control group [35]. During treatment with ATF2 inhibitors, symptoms of hepatitis in the mice regressed in parallel with a decline in activated ATF2 [35]. In addition, knockdown of both ATF2 and ATF7 induces severe abnormalities in the developing liver and heart, resulting in embryonic death in mice [84]. These findings strongly suggest the clues to potential roles of ATF2 in liver inflammation.

#### 3.3.3. Inflammatory Pain

In rats injected with complete Freund's adjuvant (CFA) to induce chronic inflammatory pain, we observed cells expressing phospho-ATF2-IR accumulating in the spinal dorsal horn. The level of p-ATF2 protein was shown to increase during the 14-day period after injection [68]. Following treatment with electroacupuncture (EA), a Chinese medical therapy for treating inflammatory pain, the ankle swelling observed in the CFA rats declined, in parallel with a decline in p-ATF2-IR-expressing cells and protein levels. These observations suggest an active role for ATF2 in regulating inflammatory pain.

#### 3.3.4. Allergic Asthma

Inflammation in allergen-induced asthma is mediated in part by release of eicosanoids [85], bioactive lipids with both anti- and proinflammatory actions in pulmonary tissues. In a mouse model of allergic asthma,* Aspergillus fumigatus* induces cPLA2*γ* (IVC PLA2 (phospholipase A2)) secretion in eosinophils and TNF-*α* expression in lung epithelial cells through macrophage activation. Underlying these effects is the recruitment of the ATF2/JUN, RELA/RELA (p65/p65), and USF1/USF2 complexes to the PLA2G4C enhancer in lung epithelial cells in response to TNF stimulation [85]. In addition, ATF2 may be an active component in autoimmune disease, vascular homeostasis, and angiogenesis [86, 87]. In Alzheimer's, Parkinson's, and Huntington's diseases, ATF2 is downregulated in the hippocampus and caudate nucleus [88], implying that ATF2 may be essential for neuronal viability and normal neurological function.

## 4. Inhibition of ATF2 and Its Applications

ATF2's role in the regulation of inflammatory mediators and diseases makes it an attractive drug target. To target the ATF2 protein in the development of new anti-inflammatory therapies requires ATF2 inhibitors of high potency and specificity. Candidate compounds applied thus far have failed tests for safety, effectiveness, and other essential characteristics.

### 4.1. Natural Compounds

Selected compounds that suppress ATF2 activity are presented in Table 2. Bichanin-A, an isoflavone, shows anti-inflammatory and antiproliferative potential through inhibition of ATF2 phosphorylation [37]. Pimaric acid from* Aralia cordata* downregulates NF-*κ*B and AP-1, leading to inhibition of ATF2 phosphorylation. This inhibits TNF-*α*-induced MMP-9 expression and the migration of human aortic smooth muscle cells (HASMCs) [38]. Nomilin, a triterpenoid present in citrus fruits, inhibits proinflammatory cytokine expression and gene expression by inhibiting ATF2 activity [39]. Piperine significantly inhibits expression of the proinflammatory mediators IL-1*β*, IL-6, TNF-*α*, GM-CSF, and IL-12P40 and also suppresses matrix metalloproteinase production [42]. The p38 kinase-specific inhibitor SB203580, the phosphatidylinositol-3-kinase-specific inhibitor LY294002, and the SAPK/JNK inhibitor JNK-interacting protein-1 (JIP-1) all inhibit ATF2 phosphorylation mediated by hepatocyte growth factor/scatter factor (HGF/SF) [36].

### 4.2. Traditional Herbs

Several traditional herbs may exert anti-inflammatory activities through inhibition of ATF2.* Schizonepeta tenuifolia* significantly suppresses LPS-induced serum levels of TNF-alpha in mice after oral administration, which is consistent with findings* in vitro* [43]. HangAmDan-B (HAD-B), a powdered mixture characterized as a folk medicine, suppresses prostaglandin E_2_ (PGE_2_) and NO production in LPS-activated macrophages. It also attenuates HCl/EtOH-induced gastritis symptoms through inhibition of JNK-ATF2 signaling [44]. Some formulations of HangAmDan-B have been applied in clinical trials. Jianpi Jiedu Recipe consists of a mixture of herbs, including* Codonopsis pilosula*,* Poria cocos*, Radices paeoniae alba, Radix bupleuri, and four others. Jianpi Jiedu Recipe is reported to reduce cyclooxygenase (COX-2) expression in a* Helicobacter pylori* (Hp) infected gastric cancer cell line, MKN45, through suppression of p38-ATF2 signaling [45]. Selected traditional herbs that inhibit ATF2 activities are presented in Table 3.

### 4.3. Gene Regulatory Factors That Inhibit ATF2 Activity

Protein factors that modulate ATF2 activity are therapeutically applicable and may be important for revealing the regulatory networks that involve proteins in this family. Krüppel-like factor 2 (KLF2), a member of the Krüppel-like factor family of zinc finger transcription factors, may exert anti-inflammatory activity through ATF2 inhibition in the nucleus [89]. Bone morphogenetic protein- (BMP-) 7 may inhibit phosphorylation of endogenous ATF2 at high doses (10 nM) [90]. PGE_2_ suppresses IL-17-induced ATF2/c-Jun transactivation and DNA binding, which is dependent on Erg-1-mediated inhibition of c-Jun expression [91].

### 4.4. Clinical Trials of ATF2 Inhibitors

Unfortunately, there are no reported clinical trials of ATF2 inhibitors. However, as inhibition of ATF2 activity does not appear to harm normal cells, systemic administration of the active agent is acceptable. Given the increasing evidence for the role of ATF2 in inflammation, clinical therapy with its specific inhibitors could be applied to treat human inflammatory diseases soon.

## 5. Summary and Perspective

Expanding data on ATF2 as a proinflammatory regulatory protein prompts us to investigate ATF2 as a molecular target in treating inflammatory disease. In particular, the strong influence of ATF2 in hepatitis virus infection is important for prevention and control of hepatitis, as well as the development of therapeutic targets. Specific inhibitors may be designed and synthesized based on structural and functional properties of ATF2. Meanwhile, natural compounds and herbal substances shown to inhibit ATF2 invite systematic study as safe and specific anti-inflammatory agents. However, their clinical utility and therapeutic index in humans have yet to be determined. The identification of newer classes of compounds with greater specificity and few side effects may augment treatments for human inflammatory diseases.

## Figures and Tables

**Table 1:** ATF family members and their functions in immune responses.

Molecule	Distribution (tissue/cell)	Functions	Reference
ATF1	(i) Ubiquitous, high in thyroid (ii) Nucleus	Regulation of malignant melanoma	[14]

ATF2	(i) Ubiquitous (ii) Nucleus and cytoplasm	See Sections 2, 3, and 4	

ATF3	(i) Ubiquitous	(i) Inhibition of MCP-1, HMGB1, and CCL4	[14, 15]
	(ii) High in placenta, pancreas, and lung	(ii) Regulation of cerebral ischemia, glial inflammation, kidney, and lung injury	[16–21]
	(iii) Nucleus	(iii) Pancreatic *β*-cells signaling, SFA/TLR4 signaling	[22, 23]

ATF4	(i) Ubiquitous (ii) Nucleus	Regulation of retinal inflammation and cytokine production in diabetes; involvement of complex formation to PKA promoter; regulation of IL-8 expression; involvement in Nrf2-ARE signaling	[24–27]

ATF5	(i) Ubiquitous (ii) High in liver, lung, adipose tissue, heart, and skeletal muscle (iii) Nucleus and cytoplasm	(i) Regulation of GR signaling pathway (ii) Involvement in various cancers	[28] [29]

ATF6	(i) Liver, serum, plasma, platelets, and cancer cells	(i) Involvement in AKT-NF-*κ*B activation signaling	[30]
	(ii) Nucleus	(ii) Induction of UPR in CF	[31]

ATF7	(i) Liver, plasma, platelets, and cancer tissues	(i) Involvement in vitamin D response in Paget's disease	[32]
	(ii) Nucleus and cytoplasm	(ii) Repressor of E-selectin/NF-ELAM1/delta-A promoter	[33]

CRE-BP*α* (CREB5)	(i) Liver, plasma, and platelet (ii) HEK293	Involvement in adipocyte differentiation	[34]

MCP-1: monocyte chemotactic protein-1, HMGB1: high mobility group box-1 protein, CCL4: macrophage inflammatory protein-1 beta, SFA: saturated fact acid, TLR4: toll-like receptor 4, CF: Cystic fibrosis, UPR: unfolded protein response, GR: glucocorticoid receptor.

**Table 2:** Natural products which inhibit ATF-2 signaling pathway.

Compound	Action target on ATF-2	Reference

Andrographolide	Suppressed NO and PGE_2_ production and ameliorated the symptoms of LPS-induced hepatitis and EtOH/HCl-induced gastritis in mice	[35]

SB203580	Inhibition of the inflammatory cytokines IL-1*β* and TNF-*α*	[36]

JIP-1	Enhances phagocytic activity, downregulation of TNF-*α*, IL-1*β*, IL-6, IFN-*γ*, IL-12, and IL-18	[36]

LY294002	Inhibition of proinflammatory cytokines NO and TNF-*α*	[36]

Biochanin-A	Inhibition of IL-6, IL-1*β*, and TNF-*α* production in RAW264.7 cells	[37]

Pimaric acid	Suppression of MMP-9 induction and migration of human aortic smooth muscle cells	[38]

Nomilin	Inhibition of proinflammatory cytokine production	[39]

Berberine	Attenuation of COX-2 overexpression during acute endotoxemia	[40]

Genistein	Inhibition of infection of cells with the New World arenavirus Pichindé (PICV)	[41]

Piperine	Reduction of proinflammatory cytokines: IL-1*β*, IL-6, TNF-*α*, GM-CSF, and MMPs	[42]

**Table 3:** Traditional herbs which inhibit the ATF-2 signaling pathway.

Plant	Action target on ATF-2	Reference
Schizonepeta tenuifolia	Reduction of LPS-induced serum levels of TNF-*α* after oral injection of mice	[43]

HangAmDan-B	Suppresses the production of PGE_2_ and NO in macrophages and ameliorates HCl/EtOH-induced gastritis	[44]

Jianpi Jiedu Recipe	Inhibition of COX-2 expression in *Helicobacter pylori* infected gastric cancer cells	[45]

## Abbreviations

ATM: Ataxia-telangiectasia-mutated protein kinase
ATF: Activating transcription factor
PG: Prostaglandin
COX: Cyclooxygenase
iNOS: Inducible nitric oxide synthase
(TNF)-*α*: Tumour necrosis factor-*α*
ERK: Extracellular signal-related kinase
TLR: Toll-like receptors
MAPK: Mitogen-activated protein kinase
NF-*κ*B: Nuclear factor-kappa B
AP-1: Activator protein-1
JNK: c-Jun N-terminal kinase
GM-CSF: Granulocyte-macrophage colony stimulating factor
PMA: Phorbol-12-myristate-13-acetate
SSc: Systemic sclerosis
ECM: extracellular matrix
KC: Keratinocyte chemoattractant
PRRs: Pattern recognition receptor family
IRAK4: IL-1 receptor-associated kinase-4
WAT: White adipose tissue
EGF: Epidermal growth factor
HFD: High-fat diet
CFA: Complete Freund's adjuvant
EA: Electroacupuncture
HASMCs: Human aortic smooth muscle cells
JIP-1: SAPK/JNK inhibitor JNK-interacting protein-1
HGF/SF: Hepatocyte growth factor/scatter factor
HAD-B: HangAmDan-B
KLF2: Krüppel-like Factor 2
BMP-7: Bone morphogenetic protein-7
CNS: Central nervous system.

## References

[1] Ferrero-Miliani L, Nielsen OH, Andersen PS, Girardin SE (2007). Chronic inflammation: importance of NOD2 and NALP3 in interleukin-1*β* generation. *Clinical and Experimental Immunology*.

[2] Ham M, Moon A (2013). Inflammatory and microenvironmental factors involved in breast cancer progression. *Archives of Pharmacal Research*.

[3] Yu T, Yi Y-S, Yang Y, Oh J, Jeong D, Cho JY (2012). The pivotal role of TBK1 in inflammatory responses mediated by macrophages. *Mediators of Inflammation*.

[4] Byeon SE, Yi Y-S, Oh J, Yoo BC, Hong S, Cho JY (2012). The role of Src kinase in macrophage-mediated inflammatory responses. *Mediators of Inflammation*.

[5] Im DS (2013). Intercellular lipid mediators and GPCR drug discovery. *Biomolecules and Therapeutics*.

[6] Youn CK, Park SJ, Lee MY (2013). Silibinin inhibits LPS-induced macrophage activation by blocking p38 MAPK in RAW 264.7 cells. *Biomolecules and Therapeutics*.

[7] Ovchinnikov DA (2008). Macrophages in the embryo and beyond: much more than just giant phagocytes. *Genesis*.

[8] Kim M-Y, Cho JY (2013). 20S-dihydroprotopanaxadiol, a ginsenoside derivative, boosts innate immune responses of monocytes and macrophages. *Journal of Ginseng Research*.

[9] Hyun B, Shin S, Lee A (2013). Metformin down-regulates TNF-alpha secretion via suppression of scavenger receptors in macrophages. *Immune Network*.

[10] Seo BS, Lee SH, Lee JE, Yoo YC, Lee J, Park SR (2013). Dectin-1 stimulation selectively reinforces LPS-driven IgG1 production by mouse B cells. *Immune Network*.

[11] Park MC, Kim D, Lee Y, Kwon HJ (2013). CD83 expression induced by CpG-DNA stimulation in a macrophage cell line RAW 264.7. *BMB Reports*.

[12] Taylor PR, Martinez-Pomares L, Stacey M, Lin H-H, Brown GD, Gordon S (2005). Macrophage receptors and immune recognition. *Annual Review of Immunology*.

[13] Kim DH, Chung JH, Yoon JS (2013). Ginsenoside Rd inhibits the expressions of iNOS and COX-2 by suppressing NF-*κ*B in LPS-stimulated RAW264.7 cells and mouse liver. *Journal of Ginseng Research*.

[14] Chen HH, Lai PF, Lan YF (2014). Exosomal ATF3 RNA attenuates pro-inflammatory gene MCP-1 transcription in renal ischemia-reperfusion. *Journal of Cellular Physiology*.

[15] Lai PF, Cheng CF, Lin H, Tseng TL, Chen HH, Chen SH (2013). ATF3 protects against LPS-induced inflammation in mice via inhibiting HMGB1 expression. *Evidence-Based Complementary and Alternative Medicine*.

[16] Wang L, Deng S, Lu Y (2012). Increased inflammation and brain injury after transient focal cerebral ischemia in activating transcription factor 3 knockout mice. *Neuroscience*.

[17] Tanaka K, Kanno T, Yanagisawa Y (2011). Bromocriptine methylate suppresses glial inflammation and moderates disease progression in a mouse model of amyotrophic lateral sclerosis. *Experimental Neurology*.

[18] Cheng C-F, Lin H (2011). Acute kidney injury and the potential for ATF3-regulated epigenetic therapy. *Toxicology Mechanisms and Methods*.

[19] Li H-F, Cheng C-F, Liao W-J, Lin H, Yang R-B (2010). ATF3-mediated epigenetic regulation protects against acute kidney injury. *Journal of the American Society of Nephrology*.

[20] Akram A, Han B, Masoom H (2010). Activating transcription factor 3 confers protection against ventilator-induced lung injury. *The American Journal of Respiratory and Critical Care Medicine*.

[21] Gilchrist M, Henderson WR, Clark AE (2008). Activating transcription factor 3 is a negative regulator of allergic pulmonary inflammation. *Journal of Experimental Medicine*.

[22] Gurzov EN, Barthson J, Marhfour I (2012). Pancreatic *β*-cells activate a JunB/ATF3-dependent survival pathway during inflammation. *Oncogene*.

[23] Suganami T, Yuan X, Shimoda Y (2009). Activating transcription factor 3 constitutes a negative feedback mechanism that attenuates saturated fatty Acid/Toll-like receptor 4 signaling and macrophage activation in obese adipose tissue. *Circulation Research*.

[24] Tacke F, Liedtke C, Bocklage S, Manns MP, Trautwein C (2005). CREB/PKA sensitive signalling pathways activate and maintain expression levels of the hepatitis B virus pre-S2/S promoter. *Gut*.

[25] Zhong Y, Li J, Chen Y, Wang JJ, Ratan R, Zhang SX (2012). Activation of endoplasmic reticulum stress by hyperglycemia is essential for Müller cell-derived inflammatory cytokine production in diabetes. *Diabetes*.

[26] Gargalovic PS, Imura M, Zhang B (2006). Identification of inflammatory gene modules based on variations of human endothelial cell responses to oxidized lipids. *Proceedings of the National Academy of Sciences of the United States of America*.

[27] Giudice A, Arra C, Turco MC (2010). Review of molecular mechanisms involved in the activation of the Nrf2-ARE signaling pathway by chemopreventive agents. *Methods in Molecular Biology*.

[28] Kochetov AV, Merkulova TI, Merkulov VM (2012). Possible link between the synthesis of GR alpha isoforms and eIF2 alpha phosphorylation state. *Medical Hypotheses*.

[29] Monaco SE, Angelastro JM, Szabolcs M, Greene LA (2007). The transcription factor ATF5 is widely expressed in carcinomas, and interference with its function selectively kills neoplastic, but not nontransformed, breast cell lines. *International Journal of Cancer*.

[30] Yamazaki H, Hiramatsu N, Hayakawa K (2009). Activation of the Akt-NF-*κ*B pathway by subtilase cytotoxin through the ATF6 branch of the unfolded protein response. *Journal of Immunology*.

[31] Kerbiriou M, Le Drévo M-A, Férec C, Trouvé P (2007). Coupling cystic fibrosis to endoplasmic reticulum stress: differential role of Grp78 and ATF6. *Biochimica et Biophysica Acta: Molecular Basis of Disease*.

[32] Teramachi J, Hiruma Y, Ishizuka S (2013). Role of ATF7-TAF12 interactions in the vitamin D response hypersensitivity of osteoclast precursors in Paget’s disease. *Journal of Bone and Mineral Research*.

[33] Pescini R, Kaszubska W, Whelan J, DeLamarter JF, van Huijsduijnen RH (1994). ATF-aO, a novel variant of the ATF/CREB transcription factor family, forms a dominant transcription inhibitor in ATF-a heterodimers. *Journal of Biological Chemistry*.

[34] Maekawa T, Jin W, Ishii S (2010). The role of ATF-2 family transcription factors in adipocyte differentiation: antiobesity effects of p38 inhibitors. *Molecular and Cellular Biology*.

[35] Shen T, Yang WS, Yi Y-S (2013). AP-1/IRF-3 targeted anti-inflammatory activity of andrographolide isolated from andrographis paniculata. *Evidence-Based Complementary and Alternative Medicine*.

[36] Recio JA, Merlino G (2002). Hepatocyte growth factor/scatter factor activates proliferation in melanoma cells through p38 MAPK, ATF-2 and cyclin D1. *Oncogene*.

[37] Kole L, Giri B, Manna SK, Pal B, Ghosh S (2011). Biochanin-A, an isoflavon, showed anti-proliferative and anti-inflammatory activities through the inhibition of iNOS expression, p38-MAPK and ATF-2 phosphorylation and blocking NF*κ*B nuclear translocation. *European Journal of Pharmacology*.

[38] Suh S-J, Kwak C-H, Chung T-W (2012). Pimaric acid from Aralia cordata has an inhibitory effect on TNF-*α*-induced MMP-9 production and HASMC migration via down-regulated NF-*κ*B and AP-1. *Chemico-Biological Interactions*.

[39] Pratheeshkumar P, Raphael TJ, Kuttan G (2012). Nomilin inhibits metastasis via induction of apoptosis and regulates the activation of transcription factors and the cytokine profile in B16F-10 cells. *Integrative Cancer Therapies*.

[40] Feng A-W, Yu C, Mao Q, Li N, Li Q-R, Li J-S (2011). Berberine hydrochloride attenuates cyclooxygenase-2 expression in rat small intestinal mucosa during acute endotoxemia. *Fitoterapia*.

[41] Vela EM, Bowick GC, Herzog NK, Aronson JF (2008). Genistein treatment of cells inhibits arenavirus infection. *Antiviral Research*.

[42] Pradeep CR, Kuttan G (2004). Piperine is a potent inhibitor of nuclear factor-*κ*B (NF-*κ*B), c-Fos, CREB, ATF-2 and proinflammatory cytokine gene expression in B16F-10 melanoma cells. *International Immunopharmacology*.

[43] Kang H, Han S-W, Hong J-W, Sohn N-W (2010). Suppression of tumour necrosis factor-*α* by Schizonepeta tenuifolia water extract via inhibition of I*κ*B*α* degradation and Jun N-terminal kinase/stress-activated protein kinase activation. *Journal of Pharmacy and Pharmacology*.

[44] Yu T, Moh SH, Kim S-B (2013). HangAmDan-B, an ethnomedicinal herbal mixture, suppresses inflammatory responses by inhibiting Syk/NF-*κ*B and JNK/ATF-2 pathways. *Journal of Medicinal Food*.

[45] Liu N-N, Wang Y, Wu Q (2011). Jianpi jiedu recipe inhibited Helicobacter pylori-induced the expression of cyclooxygenase-2 via p38MAPK/ATF-2 signal transduction pathway in human gastric cancer cells. *Zhongguo Zhong xi yi jie he za zhi*.

[46] Ozawa K, Sudo T, Soeda E-I, Yoshida MC, Ishii S (1991). Assignment of the human CREB2 (CRE-BP1) Gene to 2Q32. *Genomics*.

[47] Bhoumik A, Takahashi S, Breitweiser W, Shiloh Y, Jones N, Ronai Z (2005). ATM-dependent phosphorylation of ATF2 is required for the DNA damage response. *Molecular Cell*.

[48] Hummler E, Cole TJ, Blendy JA (1994). Targeted mutation of the CREB gene: compensation within the CREB/ATF family of transcription factors. *Proceedings of the National Academy of Sciences of the United States of America*.

[49] Vlahopoulos SA, Logotheti S, Mikas D, Giarika A, Gorgoulis V, Zoumpourlis V (2008). The role of ATF-2 in oncogenesis. *BioEssays*.

[50] Nomura N, Zu Y-L, Maekawa T, Tabata S, Akiyama T, Ishii S (1993). Isolation and characterization of a novel member of the gene family encoding the cAMP response element-binding protein CRE-BP1. *Journal of Biological Chemistry*.

[51] Gaire M, Chatton B, Kedinger C (1990). Isolation and characterization of two novel, closely related ATF cDNA clones from HeLa cells. *Nucleic Acids Research*.

[52] Zu Y-L, Maekawa T, Matsuda S, Ishii S (1991). Complete putative metal finger and leucine zipper structures of CRE-BP1 are required for the E1A-induced trans-activation. *Journal of Biological Chemistry*.

[53] Takeda J, Maekawa T, Sudo T (1991). Expression of the CRE-BP1 transcriptional regulator binding to the cyclic AMP response element in central nervous system, regenerating liver, and human tumors. *Oncogene*.

[54] Goetz J, Chatton B, Mattei M-G, Kedinger C (1996). Structure and expression of the ATFa gene. *Journal of Biological Chemistry*.

[55] Liu H, Deng X, Shyu YJ, Jian JL, Taparowsky EJ, Hu C-D (2006). Mutual regulation of c-Jun and ATF2 by transcriptional activation and subcellular localization. *The EMBO Journal*.

[56] Lau E, Ronai ZA (2012). ATF2: at the crossroad of nuclear and cytosolic functions. *Journal of Cell Science*.

[57] Ouwens DM, de Ruiter ND, van der Zon GCM (2002). Growth factors can activate ATF2 via a two-step mechanism: phosphorylation of Thr71 through the Ras-MEK-ERK pathway and of Thr69 through RaIGDS-Src-p38. *The EMBO Journal*.

[58] Gupta S, Campbell D, Derijard B, Davis RJ (1995). Transcription factor ATF2 regulation by the JNK signal transduction pathway. *Science*.

[59] Livingstone C, Patel G, Jones N (1995). ATF-2 contains a phosphorylation-dependent transcriptional activation domain. *The EMBO Journal*.

[60] Raingeaud J, Gupta S, Rogers JS (1995). Pro-inflammatory cytokines and environmental stress cause p38 mitogen-activated protein kinase activation by dual phosphorylation on tyrosine and threonine. *Journal of Biological Chemistry*.

[61] Hayakawa J, Depatie C, Ohmichi M, Mercola D (2003). The activation of c-Jun NH2-terminal kinase (JNK) by DNA-damaging agents serves to promote drug resistance via activating transcription factor 2 (ATF2)-dependent enhanced DNA repair. *Journal of Biological Chemistry*.

[62] Beier F, Taylor AC, LuValle P (2000). Activating transcription factor 2 is necessary for maximal activity and serum induction of the cyclin A promoter in chondrocytes. *Journal of Biological Chemistry*.

[63] Hayakawa J, Mittal S, Wang Y (2004). Identification of promoters bound by c-Jun/ATF2 during rapid large-scale gene activation following genotoxic stress. *Molecular Cell*.

[64] Vlahopoulos S, Zoumpourlis VC (2004). JNK: a key modulator of intracellular signaling. *Biochemistry*.

[65] Chan C-P, Chang M-C, Wang Y-J (2008). Thrombin activates Ras-CREB/ATF-1 signaling and stimulates c-fos, c-jun, and c-myc expression in human gingival fibroblasts. *Journal of Periodontology*.

[66] Pernhorst K, Herms S, Hoffmann P (2013). TLR4, ATF-3 and IL8 inflammation mediator expression correlates with seizure frequency in human epileptic brain tissue. *Seizure*.

[67] Miyata Y, Fukuhara A, Otsuki M, Shimomura I (2013). Expression of activating transcription factor 2 in inflammatory macrophages in obese adipose tissue. *Obesity*.

[68] Fang J-Q, Du J-Y, Liang Y, Fang J-F (2013). Intervention of electroacupuncture on spinal p38 MAPK/ATF-2/VR-1 pathway in treating inflammatory pain induced by CFA in rats. *Molecular Pain*.

[69] Akiyama Y, Ogawa F, Iwata Y (2009). Autoantibody against activating transcription factor-2 in patients with systemic sclerosis. *Clinical and Experimental Rheumatology*.

[70] Reimold AM, Kim J, Finberg R, Glimcher LH (2001). Decreased immediate inflammatory gene induction in activating transcription factor-2 mutant mice. *International Immunology*.

[71] Dinarello CA (2000). Proinflammatory cytokines. *Chest*.

[72] Desch M, Hackmayer G, Todorov VT (2012). Identification of ATF2 as a transcriptional regulator of renin gene. *Biological Chemistry*.

[73] Takeda K, Akira S (2004). TLR signaling pathways. *Seminars in Immunology*.

[74] Blasius AL, Beutler B (2010). Intracellular toll-like receptors. *Immunity*.

[75] Kawai T, Akira S (2010). The role of pattern-recognition receptors in innate immunity: update on toll-like receptors. *Nature Immunology*.

[76] Li X, Jiang S, Tapping RI (2010). Toll-like receptor signaling in cell proliferation and survival. *Cytokine*.

[77] Miggin SM, O’Neill LAJ (2006). New insights into the regulation of TLR signaling. *Journal of Leukocyte Biology*.

[78] Li X-Y, Green MR (1996). Intramolecular inhibition of activating transcription factor-2 function by its DNA-binding domain. *Genes and Development*.

[79] de Cesare D, Vallone D, Caracciolo A, Sassone-Corsi P, Nerlov C, Verde P (1995). Heterodimerization of c-Jun with ATF-2 and c-Fos is required for positive and negative regulation of the human urokinase enhancer. *Oncogene*.

[80] Lee J (2013). Adipose tissue macrophages in the development of obesity-induced inflammation, insulin resistance and type 2 Diabetes. *Archives of Pharmacal Research*.

[81] Kweon H-J, Suh B-C (2013). Acid-sensing ion channels (ASICs): therapeutic targets for neurological diseases and their regulation. *BMB Reports*.

[82] Skupsky J, Hu KQ (2014). Current hepatitis B treatment guidelines and future research directions. *Frontiers of Medicine*.

[83] Choi CY, Choi BH, Park GT, Rho HM (1997). Activating transcription factor 2 (ATF2) down-regulates hepatitis B virus X promoter activity by the competition for the activating protein 1 binding site and the formation of the ATF2-Jun heterodimer. *Journal of Biological Chemistry*.

[84] Breitwieser W, Lyons S, Flenniken AM (2007). Feedback regulation of p38 activity via ATF2 is essential for survival of embryonic liver cells. *Genes and Development*.

[85] Bickford JS, Newsom KJ, Herlihy J-D (2012). Induction of group IVC phospholipase A2 in allergic asthma: transcriptional regulation by TNF*α* in bronchoepithelial cells. *Biochemical Journal*.

[86] Liao H, Hyman MC, Baek AE, Fukase K, Pinsky DJ (2010). cAMP/CREB-mediated transcriptional regulation of ectonucleoside triphosphate diphosphohydrolase 1 (CD39) expression. *Journal of Biological Chemistry*.

[87] Licht AH, Pein OT, Florin L (2006). JunB is required for endothelial cell morphogenesis by regulating core-binding factor *β*. *Journal of Cell Biology*.

[88] Pearson AG, Curtis MA, Waldvogel HJ, Faull RLM, Dragunow M (2005). Activating transcription factor 2 expression in the adult human brain: association with both neurodegeneration and neurogenesis. *Neuroscience*.

[89] Fledderus JO, van Thienen JV, Boon RA (2007). Prolonged shear stress and KLF2 suppress constitutive proinflammatory transcription through inhibition of ATF2. *Blood*.

[90] Hu MC, Wasserman D, Hartwig S, Rosenblum ND (2004). p38MAPK acts in the BMP7-dependent stimulatory pathway during epithelial cell morphogenesis and is regulated by Smad1. *Journal of Biological Chemistry*.

[91] Faour WH, Alaaeddine N, Mancini A, Qing WH, Jovanovic D, Di Battista JA (2005). Early growth response factor-1 mediates prostaglandin E2-dependent transcriptional suppression of cytokine-induced tumor necrosis factor-*α* gene expression in human macrophages and rheumatoid arthritis-affected synovial fibroblasts. *Journal of Biological Chemistry*.

